# A LIDAR-Compatible, Multichannel Raman Spectrometer for Remote Sensing of Water Temperature

**DOI:** 10.3390/s19132933

**Published:** 2019-07-03

**Authors:** Andréa de Lima Ribeiro, Christopher Artlett, Helen Pask

**Affiliations:** 1 MQ Photonics Research Centre, Department of Physics and Astronomy, Macquarie University, Sydney 2109, Australia; 2 Defence Science & Technology Group, Maritime Division, Eveleigh 2015, Australia

**Keywords:** Raman spectroscopy, remote sensing, water, temperature, natural waters, LIDAR

## Abstract

The design and operation of a custom-built LIDAR-compatible, four-channel Raman spectrometer integrated to a 532 nm pulsed laser is presented. The multichannel design allowed for simultaneous collection of Raman photons at two spectral regions identified as highly sensitive to changes in water temperature. For each of these spectral bands, the signals having polarization parallel to (∥) and perpendicular to (⟂), the excitation polarization were collected. Four independent temperature markers were calculated from the Raman signals: two-colour(∥), two-colour(⟂), depolarization(A) and depolarization(B). A total of sixteen datasets were analysed for one ultrapure (Milli-Q) and three samples of natural water. Temperature accuracies of ±0.4 °C–±0.8 °C were achieved using the two-colour(∥) marker. When multiple linear regression models were constructed (linear combination) utilizing all simultaneously acquired temperature markers, improved accuracies of ±0.3 °C–±0.7 °C were achieved.

## 1. Introduction

Water temperature is an important parameter in aquatic environments, directly influencing the water column structure and allowing for the investigation of physical and biological processes such as ocean currents, heat exchange, pycnocline depths, geostrophic flow, detection of upwelling systems, and primary productivity. Researchers rely on both traditional in situ sampling methods and remote sensing techniques to gain water temperature information.

Traditional methods, such as thermometers and temperature probes deployed from ships and vessels, allow for acquisition of depth-resolved highly accurate data; operational logistics, however, are complex, with information collected at a limited number of sampling stations and not compatible with meso and macroscale processes at oceanic and coastal zones [1]. Efforts to overcome these issues resulted in development of new technologies to remotely monitor the oceans, for instance, satellite sensors and LIDAR (Light Detection and Ranging) methods.

Remote sensing methods retrieve data from an object without direct interaction by using sensors to detect electromagnetic, acoustic or electrical signals [2,3]. Infrared satellite sensors, such as the Advanced Very High Resolution Radiometer (AVHRR), retrieve signals spontaneously emitted by the oceans and are currently the main contributors for water temperature monitoring programs, providing a synoptic view of the oceans at larger scales than in situ measurements [4]. However, infrared radiation undergoes pronounced absorption in water and only signals emitted by the first micrometers of water column are retrieved by the sensors, rendering the collection of subsurface information ineffective. Besides depth limitations, data acquisition is restricted to areas without cloud coverage and requires validation with in situ data for increased accuracies. Recent AVHRR accuracy estimations indicates errors of up to ±2.0 °C in temperature predictions at the coast and ±1.0 °C for oceanic zones [5].

Limitations of both in situ and satellite methods expose a technological gap to be filled by a remote sensing technique able to provide depth-resolved temperature data at scales not covered by either of the abovementioned, such as LIDAR methods. LIDAR methods in oceanography include active and passive remote sensing techniques where signals in the visible or near-infrared range emitted by a target are retrieved by a sensor and interpreted to derive depth-resolved information. Active LIDAR equipment requires monochromatic short-pulsed light as an excitation source, which is transmitted down the water column interacting with molecules and other optically active constituents. By considering the arrival time of returning excitation photons and/or photons at different frequencies, it is possible to assess depth-resolved environmental information such as bathymetry, fluorescence from optical constituents, and, ultimately, water temperature. In this regard, optical methods retrieving backscattered light such as Raman or Brillouin spectroscopy have the potential to be coupled to LIDAR technologies and ultimately provide real-time reliable data of subsurface water temperature for regional and global studies [6,7,8,9,10,11]. Our focus in this paper is on applying Raman methods, as they are most amiable to the development of a compact and affordable instrument.

Raman spectroscopy (RS) is a technique based on the inelastic scattering of an incident photon by a molecule, resulting in photons being scattered with a shift in frequency relative to the excitation source [12]. In the liquid state, water molecules exhibit Raman active modes associated with translational, librational, bending, and stretching forces [13,14]. These Raman active modes present temperature-dependent behavior, the origin of which is somewhat contentious. The OH stretching band is the most prominent feature in the water Raman spectrum, extending from 2900 to 3900 cm^−1^ and exhibiting an isosbestic point at which signal intensities are insensitive to changes in temperature [13,15]. Researchers have proposed various techniques for using the temperature dependence of Raman spectra to predict water temperature [8,16,17,18,19]. Our approach has been guided by our longstanding goal of developing a compact and affordable instrument. Further, we seek to exploit both the frequency and polarization dependence of Raman spectra.

**Figure 1:**
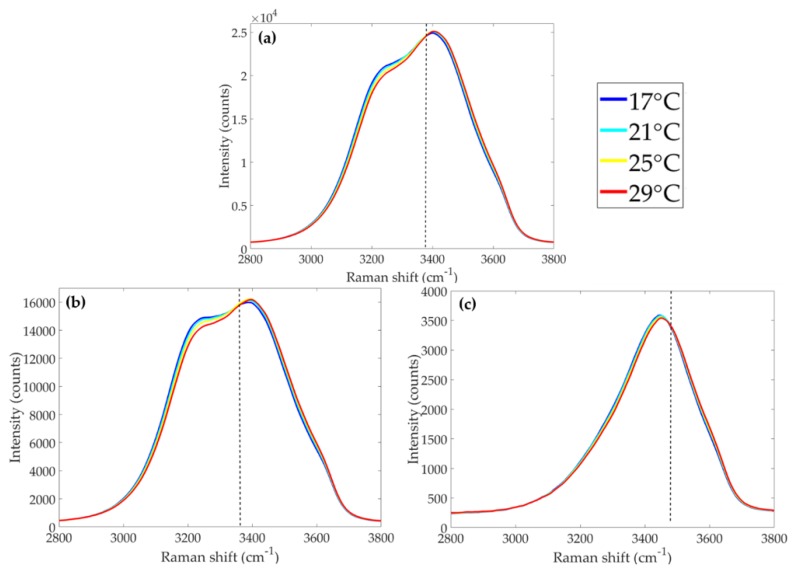
Temperature-dependent Raman spectra from ultrapure (reverse osmosis) water. (**a**) unpolarized spectra; (**b**) parallel-polarized spectra; (**c**) perpendicularly-polarized spectra. Isosbestic points are indicated by a dashed line.

Polarized RS reveals different shapes and intensities for Raman signals according to their state of polarization relative to that of the excitation laser. The unpolarized and polarized Raman spectra presented in Figure 1 were measured using a dispersive commercial Raman spectrometer (Enwave EZRaman-I, integrated with a 532 nm CW laser), with polarizing filters inserted as required. “Unpolarized” refers to all Raman photons, regardless of their state of polarization (Figure 1a); “parallel-polarized” refers to photons scattered having the same state of polarization as the excitation sources (Figure 1b); and “perpendicularly-polarized” implies Raman photons being scattered with polarization state orthogonal to that of the excitation light (Figure 1c). Parallel-polarized components exhibit higher signal intensities than perpendicularly polarized signals, in conformity with the tetrahedral geometry of water molecules [20] (Figure 1b,c).

Regardless of the polarization state, the isosbestic point marks an inversion of Raman signal behavior: for shifts below (above) the isosbestic point, higher intensities are associated with lower (higher) temperatures. Following the first studies correlating the temperature-dependent behavior of water Raman signal around the OH-stretching band, temperature markers were proposed for unpolarized and polarized water Raman spectra, known respectively as two-colour and depolarization ratios.

Two-colour temperature markers, also referred as “two-colour ratios”, have been most widely used in Raman temperature prediction studies [9,10,11,21]. In most studies, full, unpolarized water Raman spectra are decomposed in two or more Gaussian curves and a ratio is taken of the areas under these Gaussians or some other feature such as their spectral widths. More recently, a different approach for the two-colour method was reported in References [11,22] which did not require spectral decomposition. Raman signals were integrated within channels on both sides of the isosbestic point and temperature markers were calculated based on the ratio of integrated signal intensities for each channel. By using two-colour markers calculated from channel integrations, accuracies as high as ±0.1 °C were achieved for ultrapure water (Reverse-Osmosis) and ±0.2 °C for natural water samples [23] measured in laboratory.

Depolarized temperature markers have been calculated as ratios between the perpendicularly-polarized and parallel-polarized Raman signal intensities within a band of wavelengths. In water, these ratios exhibit a linear temperature-dependent behavior and can be used for temperature predictions. In [19], polarized Raman components were acquired from a saline solution (NaCl 40%) and used for estimating depolarization markers, achieving theoretical accuracies of ±0.5 °C for temperature predictions. Later, the same temperature prediction accuracies of ±0.5 °C were achieved when collecting Raman spectra from water excited by a 470 nm laser [24]. Many Raman spectrometers, including the one used to acquire Figure 1, do not allow for simultaneous acquisition of orthogonally-polarized spectral components. Accordingly, the use of depolarization markers has not been investigated in recent years.

Raman spectroscopy has proven to be an effective technique for determining water temperature in the laboratory with high accuracies of up to ±0.1 °C and ±0.5 °C using two-colour or depolarization markers, respectively [11,24]. The reports in [23,25,26] propose the possibility of measuring subsurface water temperature using Raman spectroscopy in combination with LIDAR methods, collecting time-resolved Raman signals in channels selected by optical filters. This is the ultimate goal of our research project. Our arrangement is LIDAR-compatible in that it uses a short pulse (< 2 ns) excitation laser and fast photomultipliers, and is compatible with underwater, surface, and possibly airborne platforms. However, the work presented here is at an early stage.

In this work, we report a custom-built multichannel Raman spectrometer incorporating a short- pulsed excitation source, optical filters, and fast detector, and we study small volumes of ultrapure (Milli-Q) and natural waters which were collected from Sydney Harbor. Prior to implementing LIDAR methods in large volumes, we need to evaluate and optimize the optical design of the multichannel spectrometer and methods for analyzing the collected signal. Our multichannel spectrometer enabled simultaneous collection of parallel and perpendicularly-polarized Raman signals, enabling the investigation of both two-colour and depolarization temperature markers. Root Mean Squared Temperature Error (RMSTE) values were estimated for temperature predictions performed by both types of markers and the sensitivity of each marker (% change per °C) was also evaluated. Lastly, we propose a new, innovative, linear combination method which uses both two-colour and depolarization markers for enhanced temperature predictions.

## 2. Materials and Methods

### 2.1. Spectrometer Design

The excitation source for the multichannel Raman spectrometer was a 532 nm Nd:YAG, passively Q-switched, pulsed laser (Innolight µFlare) having 25 μJ per pulse, 0.9 ns pulse duration full width at half maximum (FWHM), and pulse repetition rate of 4.5 kHz. The water samples used in the study were ultrapure (Milli-Q) and three natural water samples collected from Sydney Harbour at different times. These were analyzed within a few hours of collection.

**Figure 2:**
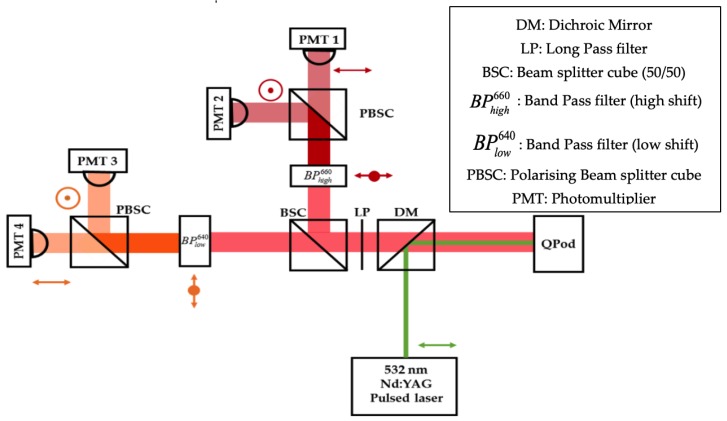
Experiment setup.

**Figure 3:**
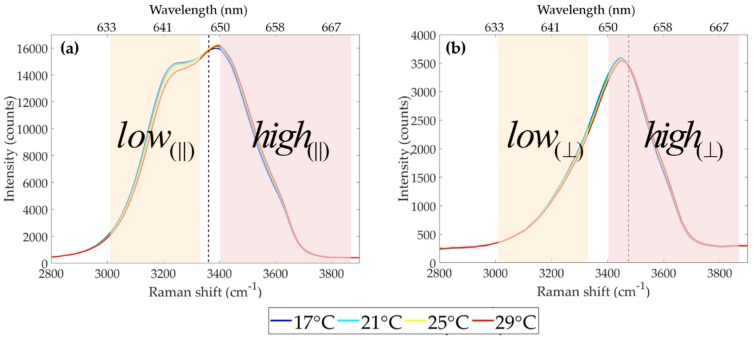
Band pass filter transmissions superimposed on **(a)** parallel and **(b)** perpendicularly-polarized Raman spectra. Low and high channels are indicated by shaded areas.

Our experimental setup is shown in Figure 2. A water sample was placed inside a temperature-controlled cuvette holder (QPod2e, accurate to ±0.15 °C) and its temperature was varied from 18 °C to 40 °C (stepping every 2 °C). The oscilloscope was triggered by inserting a glass window in the laser path, before it was coupled into the spectrometer, deflecting ~4% of the incident beam towards a photodiode connected to the oscilloscope. Excitation photons (532 nm) were reflected by a Dichroic Mirror (DM, reflectivity R~94% at 532 nm, transmission T~98% between 620 and 670 nm) and focused into the water sample by a converging lens (f = 70.0 mm). Red-shifted Raman photons scattered by the sample passed through a Long Pass filter (LP, R~99.9% at 532 nm and T~98% at 620-670 nm) in order to reject most Rayleigh-scattered photons. The Stokes photons were split into two directions, by a non-polarizing beam splitting cube (BSC), one beam then passing through $BP_{low}^{640}$ (Semrock LD01-640/8-25, central wavelength: 640 nm, band-pass: 12.9 nm at FWHM), and the other through $BP_{high}^{660}$ (Semrock FF01-660/13-25 nm, central wavelength: 660 nm, and band-pass: 20.2 nm at FWHM). The choice of these filters was constrained by commercial availability and total spectral widths at FWHM were 315 cm^−1^ and 463 cm^−1^ for low and high shift channels, respectively. Their spectral pass bands are shown superimposed on the polarized Raman spectra in Figure 3. These filters had high rejection (OD > 5) outside their pass bands.

Each beam was then divided into two polarized components by a polarization beam splitting cube (PBSC), prior to detection by a fast Photomultiplier (Hamamatsu H10721-20, rise time ~1 ns) coupled to a converging lens (f = 25.0 mm) to focus the backscattered Raman photons into the detectors aperture. The PMT gain values were set around 700 V for all channels, well below the maximum gain allowed by our PMTs (900 V). Raman signal intensities were simultaneously registered by a multichannel oscilloscope (Tektronix DPO4104B), each being an average of 512 pulses. Signal-to-noise (SNR) ratios were calculated for each channel according to Equation (1).

(1) $$SNR=\frac{\int {\mathrm{Signal}}_{\left(\mathrm{FWHM}\right)}}{\int {\mathrm{Noise}}_{\left(\mathrm{FWHM}\right)}}$$

where $\int {\mathrm{Signal}}_{\left(\mathrm{FWHM}\right)}$ represents the integrated Raman signal pulse over the full width of half maximum (FWHM); and $\int {\mathrm{Noise}}_{\left(\mathrm{FWHM}\right)}$ refers to the integration of the noise signals over the FWHM. For each water sample, three independent acquisitions were performed for each temperature, hence three sets of two-colour and depolarization markers could be calculated for each temperature. Aiming to increase robustness, the markers calculated from the independent datasets were averaged, giving origin to a new (fourth) dataset for each temperature marker hereafter referred as the “average markers dataset.

Table 1 shows a list with information regarding all spectral channels collected by this setup and correspondent nomenclatures adopted in this study.

### 2.2. Temperature Markers

**Figure 4:**
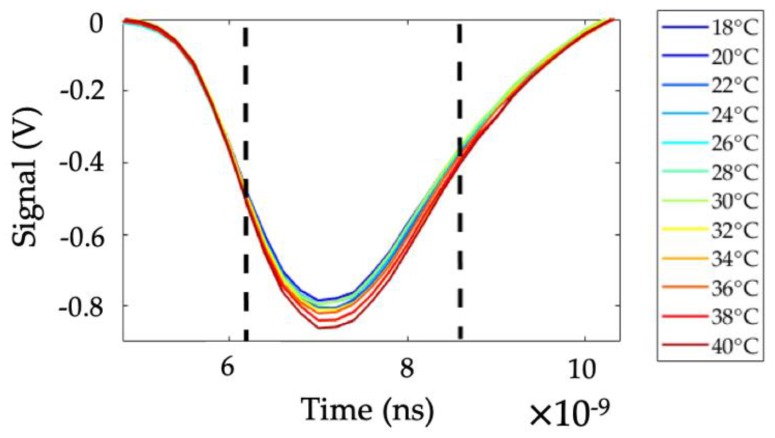
A typical set of signals (channel 1), recorded for different temperatures and showing the area over which the signals were integrated.

Each pulse registered by the oscilloscope was integrated over a range of 2.0 ns (10 data points), as indicated in Figure 4, using the Trapezoidal rule. Integrated signals for each channel were used to calculate four temperature markers as expressed by Equations (2)–(5).

(2) $$Two-colour(∥)=\frac{I_{∥}^{high}}{I_{∥}^{low}}$$

(3) $$Two-colour(⊥)=\frac{I_{⊥}^{high}}{I_{⊥}^{low}}$$

(4) $$Depolarisation(A)=\frac{I_{⊥}^{high}}{I_{∥}^{low}}$$

(5) $$Depolarisation(B)=\frac{I_{⊥}^{low}}{I_{∥}^{high}}$$

### 2.3. Predicting Temperatures

Linear regression models were constructed from the relationships between temperature markers and reference temperature, and their coefficients (*gradient*, *intercept*) were obtained for each marker analysis. These coefficients were rearranged in order to calculate a new set of temperatures dependent on the markers, hereafter called “predicted temperatures” (Equation (6)).

(6) $$T_{predicted}=(\mathrm{gradient}\;\times \;\mathrm{marker})\;+\;\mathrm{intercept}$$

where $T_{predicted}$ represents the predicted temperature estimated by a two-colour or depolarization ratio (marker). Plotting these predicted temperatures against the measured reference temperatures enabled RMSTE values to be calculated; these RMSTE values provided our measure of temperature prediction accuracy.

### 2.4. Marker Sensitivity to Temperature

Marker sensitivities were also estimated for an ultrapure water sample, representing the percentage change in the marker values per °C. For natural water samples variations in the markers values may be associated with the presence of fluorescence from other optically active components in water, as reported in Reference [23], hence not representing the markers sensitivity to temperature only.

As described in Reference [11], the use of mean-scaled temperature markers is appropriate for sensitivity calculations, and accordingly each temperature marker was divided by the mean of all markers within a set of temperature measurements (Equation (7)). Sensitivity information was extracted from the slope calculated for the linear model correlating mean-scaled markers and their respective temperatures. The use of mean-scaled markers also enables comparison between different types of markers calculated for a given water sample, determining which markers are associated with higher sensitivities.

(7) $$\mathrm{Mean}\text{-}\mathrm{scaled}\;\mathrm{marker}\;\mathrm{sensitivity}=\frac{d(\mathrm{marker})}{dT}\frac{1}{mean(\mathrm{marker})}$$

### 2.5. Linear Combination Method (LC)

Multiple linear regression (or linear combination) is a multivariate analysis method used for modelling linear relationships between two or more independent variables (in this study, temperature markers) and a set of dependent measurements (reference temperatures). Our spectrometer design enabled simultaneous collection of signals at all channels, allowing for combining temperature markers into one model to enhance the accuracy of temperature predictions Equation (8).

(8) $$T_{predicted}=\beta_{0}+\beta_{1}\times two-colour(∥)+\beta_{2}\times two-colour(⊥)+\beta_{3}\times depol(A)+\beta_{4}\times depol(B)+\varepsilon$$

where β_0_ is an independent term, β_1–_β_4_ are calibration terms generated by the model and correlated with each marker, and ε are the residual errors.

## 3. Results and Discussion

### 3.1. Milli-Q (ultrapure) Water Analysis

Temperature markers calculated from Raman signals scattered by a Milli-Q (ultrapure) water samples were analysed in order to determine sensitivities, percentage errors in the markers associated with SNRs and the accuracy with which temperature could predicted (RMSTEs). Due to the absence of other signals overlapping with the Raman peak, these values should indicate the maximum performances that could be achieved by our RS in laboratory experiments. A summary with the main results found for ultrapure water analysis is shown in Table 2.

**Figure 5:**
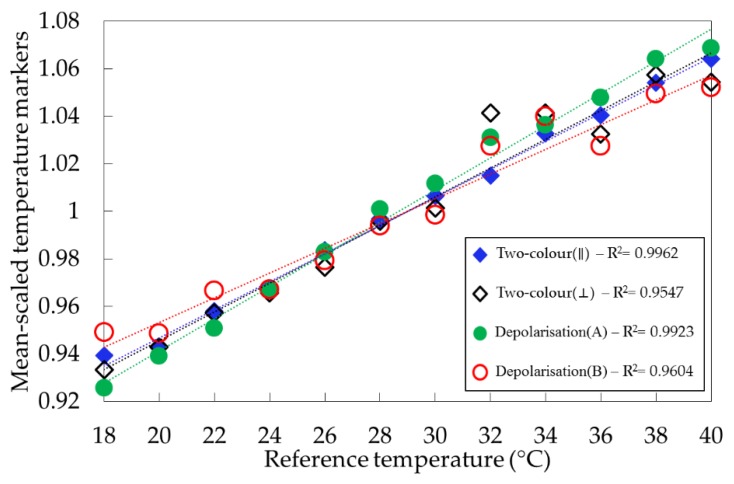
Mean-scaled temperature markers for Milli-Q water.

The response of each marker to changes in temperature was investigated by comparing their mean-scaled temperature markers (Figure 5), and the sensitivities were extracted from the slope of the linear relationships between mean-scaled markers and their respective temperatures (Table 2).

Similar sensitivities were found for all temperature markers calculated from the ultrapure water sample, varying from 0.52%/°C (depolarization(B)) to 0.68%/°C (depolarization(A)). R^2^ values were found to be poor for two-colour(⟂)) and depolarization(B) when compared with other temperature markers and it can be seen in Table 2 that these were also the markers that had higher percentage errors. The sensitivities are somewhat lower than the values around 1%/°C for two-colour markers calculated from full unpolarized Raman spectra reported by the authors of [8,9,21]. We attribute this to our use of spectral channels and the inevitable trade-off between sensitivity and signal intensity. These trade-offs were explored in [11], where the authors simulated the impact of channel widths on two-colour markers sensitivities calculated from unpolarized Raman signals. Analysis of an ultrapure water sample (Reverse-Osmosis) showed a systematic decrease in the marker sensitivities when increasing the spectral channels widths for Raman signal acquisition. In that simulation, sensitivities of 0.52%/°C were reported for channels of 300 cm^−1^ width, and an optimal channel width of 200 cm^−1^ was suggested.

Accuracies found for Milli-Q water analysis varied from ±0.4 °C to ±2.1 °C, as shown in Table 2. RMSTEs were more aligned with the percentage errors calculated for each marker, derived from channels SNRs, than with the markers’ sensitivities. The best RMSTEs of ±0.4 °C were found for two-colour(∥) analysis, and are comparable to the values of ±0.4 °C reported in other LIDAR-compatible RS reports [11,22].

### 3.2. Natural Water Analysis

RMSTEs, sensitivities, and percentage errors calculated for all temperature markers retrieved from natural water samples are shown in Table 3. The data is compiled from 12 datasets, as detailed in Section 2.1. We first start by considering the markers sensitivities in natural waters. All markers exhibited sensitivities lower than the ones found for Milli-Q waters, which can be explained by the presence of other optically active constituents in natural waters. Issues regarding fluorescence from chlorophyll-a and Dissolved Organic Matter overlapping with the Raman peak when excitation is at 532 nm and temperature predictions have already been addressed in [23,27]. Unwanted fluorescence signals contribute to the overall signal counts leading to higher SNR (and therefore lower percentage errors in the temperature markers), which can be seen in nearly all-natural water samples under analysis (Table 3) when compared with the Milli-Q water results (Table 2). Thus, the percentage errors determined for natural waters need to be interpreted cautiously, and the values in Table 2 may be more meaningful.

Higher accuracies (i.e., lower RMSTEs) were found when using two-colour(∥) markers for all-natural water samples, with RMSTEs ranging from ±0.4 °C to ±0.9 °C. This is consistent with the findings for Milli-Q water. The RMSTE values are also similar, and we note that the sensitivities found for natural water samples are within 15% of the Milli-Q water values. The accuracies obtained using the two-colour(⟂) marker were more variable, with RMSTEs ranging from ±0.9 °C to ±2.6 °C. There was more variation in the marker sensitivity between samples, with the values differing from the Milli-Q results by as much as 50%. The higher RMSTEs were associated with lower sensitivity, which suggests this marker is less immune to the presence of fluorescing constituents.

This was the first time, to our knowledge, that two-colour markers were calculated from polarized Raman signals selected by optical filters. The accuracies achieved using the two-colour(∥) markers (±0.4 °C –±0.9 °C) are broadly consistent with the accuracies reported in Reference [23], where RMSTEs within the range of ±0.3 °C–±1.0 °C were predicted for natural water samples based on the full unpolarized Raman spectra collected by a commercial RS, integrating Raman signals in channels of 200 cm^−1^ width. Strategies were presented in Reference [23] which corrected for fluorescence, and reduced the RMSTEs to ±0.2 °C–±0.5 °C. We anticipate the “correction by temperature marker values” method presented in Reference [23] could be implemented in the multichannel RS described here. We hope to achieve better accuracies with our LIDAR-compatible, multichannel RS with the use of custom-built Band Pass filters with smaller bandwidths.

Next, we consider the use of depolarization temperature markers. The RMSTEs varied widely from ±0.8 °C to ±8.1 °C (Table 3), and it was not possible to infer which of the depolarization markers had the better performance. For each marker, the smaller RMSTEs were associated with higher sensitivity. There is limited literature with which to compare our RMSTEs based on depolarization markers. As explained in Reference [24], depolarization markers are traditionally calculated from signals at different state of polarizations but within the same spectral band (unlike the present study), exhibiting the advantage of not being impacted by fluorescence signals and differential attenuation when propagating in water. The authors of Reference [24] determined water temperature from polarized Raman spectra acquired by using a 470 nm dye laser as excitation, achieving accuracies of up to ±0.5 °C. Based on our observations, the depolarization markers predict temperatures less accurately than the two-colour ratios. It is possible, however, that in the future field studies the benefits outlined by Leonard [24] might become significant and a better selection of filters excluding the temperature-insensitive points for the depolarized Raman band identified by the authors of [28].

### 3.3. Enhancing the Accuracy of Temperature Predictions Using Linear Combination Methods

While the two-colour(∥)markers clearly enabled the most accurate prediction of temperature for all the water samples investigated here, it is equally clear that the other markers also exhibit temperature dependence, albeit to a lesser degree. Accordingly, we now apply the linear combination method described in Section 2.1 to our four water samples. RMSTE values of temperature predictions for natural and Milli-Q water samples after LC are shown in Table 4.

RMSTEs after the LC method exhibited average improvements of 30% relative to the best RMSTE obtained using a single marker, with final accuracies after LC equal or better than ±0.5 °C for all samples. The effectiveness of the LC method is largely due to the nature of the multiple linear regression, where lower weightings (β values) are associated with markers that are less useful. Allied with simultaneous signal collection by our spectrometer, LC, was effective in extracting temperature-related information from all markers and maximizing the accuracies of temperature predictions for all water samples.

## 4. Conclusions

In this paper we presented a custom-built multichannel Raman spectrometer, operating with a 532 nm pulsed laser and commercial optical filters collecting polarized signals on spectral regions of interest for temperature predictions in natural waters. The design is LIDAR-compatible, employing (1) a pulsed laser source of ≤ 2 ns full-width at half maximum, desirable to achieve a depth resolution better than 0.5 m; (2) collection of Raman signals from optical channels through the use of Band Pass filters; (3) fast, sensitive detection by photomultipliers.

This was the first time that polarized Raman signals collected from different spectral channels were simultaneously selected by optical filters and used effectively for temperature prediction, achieving accuracies as high as ±0.4 °C with minimal processing. The innovative 4-channel design of our equipment enabled 4 temperature-dependent markers to be utilized. It also allowed for the use of linear combination methods, which significantly enhanced the accuracy of temperature predictions. Temperature accuracies were closely associated with the sensitivities of each marker, and the percentage error within each marker derived from signal-to-noise ratios at the channels of Raman signal collection.

The fact that our setup is compatible with LIDAR technologies and allows for LC methods to be used represents a major advance for using Raman spectroscopy as a reliable technique able to determine natural water temperature with accuracies higher than current remote sensing tools. In the next stages of our project, we will apply this methodology to a large number of water samples (> 30), so that the accuracies of temperature predictions can be analysed using Gaussian statistics (e.g., ANOVA method). The relatively small number of samples and Raman signals collected in this work did not permit the use of such statistics. Future work includes evaluating LIDAR capability by probing large volumes of water in a suitable cell or *in situ*. This will enable us to answer key questions related to the ultimate usefulness of our methods, as well as providing information to guide future LIDAR calculations.

## Figures and Tables

**Table 1:** Nomenclature adopted for each spectral channel.

Channel Number	Polarization State	Band Pass Filter	Nomenclature	Typical SNR Values
1	Parallel	$BP_{high}^{660}$	$I_{∥}^{high}$	$1.9\times 10^{4}$
2	Perpendicular	$BP_{high}^{660}$	$I_{⊥}^{high}$	$1.6\times 10^{4}$
3	Perpendicular	$BP_{low}^{640}$	$I_{⊥}^{low}$	$2.3\times 10^{4}$
4	Parallel	$BP_{low}^{640}$	$I_{∥}^{low}$	$7.7\times 10^{4}$

**Table 2:** RMSTEs, sensitivities, and the absolute percentage errors in each marker for a Milli-Q water sample. Data in brackets is based on the analysis of 4 datasets; data without brackets is based on the “average markers” dataset. Refer to Section 2.1. for details.

Temperature Marker	RMSTE (±°C)	Sensitivity (%/°C)	Absolute Percentage Error in Marker (%)
**Two-colour (∥)**	0.4 [0.4–0.7]	0.59	0.00093
**Two-colour (⟂)**	1.5 [1.5–1.7]	0.61	0.0035
**Depolarization (A)**	0.8 [0.8–1.0]	0.68	0.0021
**Depolarization (B)**	1.8 [1.4–2.1]	0.52	0.0023

**Table 3:** RMSTEs, sensitivities, and the absolute percentage errors in each marker for natural water sample analysed by two-colour markers. Data in brackets is based on the analysis of 4 datasets; data without brackets is based on the “average markers” dataset. Refer to Section 2.1. for details.

		Temperature Markers			
		Two-Colour(∥)	Two-Colour(⟂)	Depolarization(A)	Depolarization(B)
**Natural 1**	RMSTE (±°C) (Range)	0.4 (0.4–0.6)	2.6 (2.3–2.6)	1.6 (1.6–1.7)	2.1 (2.1–2.5)
	Sensitivity (%/°C)	0.50	0.30	0.48	0.30
	Marker percentage error (%)	0.00098	0.0026	0.0019	0.0017
**Natural 2**	RMSTE (±°C) (Range)	0.7 (0.5–0.7)	1.3 (1.0–1.3)	1.4 (0.8–3.4)	1.1 (1.1–2.2)
	Sensitivity (%/°C)	0.57	0.57	0.59	0.56
	Marker percentage error (%)	0.00089	0.00276	0.00179	0.00187
**Natural 3**	RMSTE (±°C) (Range)	0.8 (0.8–0.9)	0.9 (0.9–1.7)	6.5 (5.6–8.1)	2.6 (2.5–2.7)
	Sensitivity (%/°C)	0.53	0.49	0.25	0.78
	Marker percentage error (%)	0.00084	0.0024	0.0017	0.0016

**Table 4:** RMSTE improvement after linear combination (LC) methods.

Sample	Best RMSTE for single marker [Range for all markers] (±°C)	Best RMSTE after LC [Range] (±°C)	Improvement due to LC (%)
**Milli-Q water sample**	0.4 [0.4–2.1]	0.3 [0.3–0.5]	25
**Natural sample 1**	0.4 [0.4–2.6]	0.3 [0.3–0.5]	25
**Natural sample 2**	0.5 [0.5–3.4]	0.4 [0.3–0.5]	20
**Natural sample 3**	0.8 [0.8–8.1]	0.5 [0.5–0.7]	38
